# Cumulative experience improves the procedures of mechanical thrombectomy

**DOI:** 10.1186/s12883-022-02562-z

**Published:** 2022-01-25

**Authors:** Chengfang Liu, Yukai Liu, Zhongyuan Li, Pengyu Gong, Zhaohan Xu, Junshan Zhou, Weidong Zhang

**Affiliations:** 1 Department of Neurology, Nanjing First Hospital, Nanjing Medical University, No. 68 Changle Road, Nanjing, Jiangsu Province 210006 People’s Republic of China; 2 Department of Radiology, Nanjing First Hospital, Nanjing Medical University, No. 68 Changle Road, Nanjing, Jiangsu Province 210006 People’s Republic of China

**Keywords:** Experience, Mechanical thrombectomy, Large vessel occlusion stroke, Recanalization, Annual

## Abstract

**Background:**

Mechanical thrombectomy has been widely performed for large vessel occlusion stroke. The present study aimed to determine whether cumulative experience could improve thrombectomy outcomes.

**Methods:**

In this retrospective single-center analysis, patients who underwent mechanical thrombectomy with the Solitaire stent in 3 years from 25 April 2015 were enrolled in the current study. Patients’ characteristics, durations of admission and treatment, recanalization rates, clinical outcomes, and hemorrhage transformation rates were compared among the 3 years. Logistic analysis was used to analyze the independent correlation of the years and procedural outcomes.

**Results:**

A total of 222 patients underwent mechanical thrombectomy in the 3 years: 50 in the first year, 68 in the second year, and 104 in the third year. Door-to-puncture time (*P* < 0.001) and puncture-to-recanalization time (*P* = 0.033) decreased significantly among the 3 years, while successful recanalization rates increased (*P* = 0.001). Logistic regression analysis showed an independent increase in the successful recanalization rates in the second year and third year (*P* = 0.020, P = 0.001) as compared to that in the first year.

**Conclusions:**

Cumulative experience might improve the procedures of mechanical thrombectomy. The current findings suggested a potential benefit for centralization in the treatment of large vessel occlusion stroke.

## Background

Since the landmark trials were sequentially published from 2015, mechanical thrombectomy has become the key treatment for acute large vessel occlusion stroke [1, 2]. Previous studies established a correlation between site volume and thrombectomy outcomes. A retrospective analysis on a national database found less mortality rates after thrombectomy in patients transferred to the high-volume hospitals than the low-volume [3]. A multicenter registry of stent-retriever thrombectomy also found that short duration of the procedure and improved outcome were seen in high volume sites [4]. These findings implied an intuitive impact of operators’ and centers’ experience on the effect and safety of mechanical thrombectomy [5]. Based on this evidence, a recent multi-society consensus requires a minimum of 50 procedures annually for centers that perform thrombectomy [6]. Accumulating evidence from randomized trials and recommended guidelines showed an increasing trend of thrombectomy procedures performed worldwide [7]. However, the influence of cumulative practice, especially beyond the recommended minimum annual procedures, on the effect of thrombectomy is not yet clarified. Although a learning curve was widely accepted, the steep or smooth trend is controversial [8, 9]. Thus, we employed a retrospective analysis based on the prospectively collected stroke registry of the Nanjing First Hospital. The first procedure of mechanical thrombectomy was performed in 2015 following the updated guidelines on endovascular treatment [1]. The group consisted of three senior neuroradiologists with prior experience of interventional procedures, such as carotid artery stenting and intra-artery thrombolysis but not mechanical thrombectomy. Herein, we investigated the thrombectomy procedures of three consecutive years from the first case and explored whether there is a continuous improvement trend.

## Methods

The first procedure was undertaken on April 25, 2015 and the 3 years lasted up to April 24, 2018. All patients who underwent mechanical thrombectomy with the Solitaire stent for large vessel occlusion stroke in these 3 years were enrolled from our single-center stroke registry. Patients were categorized into three consecutive annual groups according to the procedure date: the first-year group (April 25, 2015 to April 24, 2016), the second-year group (April 25, 2016 to April 24, 2017), and the third-year group (April 25, 2017 to April 24, 2018). Baseline characteristics including medical history, laboratory test results, stroke severity and diagnosis, details of procedure such as recanalization status, and clinical outcomes were collected. The admission stroke severity was accessed using the National Institute of Health Stroke Scale (NIHSS) score. The recanalization status was measured by the modified thrombolysis in cerebral infarction (mTICI) scale and successful recanalization was defined as a score of 2b or 3 [10]. There is a new metric for thrombectomy devices named the first pass effect (FPE). It means achieving complete revascularization of the large vessel occlusion and its downstream territory in single pass or use of the device without rescue therapy [11]. The time points of stroke onset, admission to hospital (door), groin puncture, and first recanalization (or end of the procedure for patients failed to acquire successful recanalization) were recorded. A good outcome was defined as a modified Rankin scale (mRS) score 0–2 at 90 days after the stroke onset. Repeated head CT was performed within 24 h to ascertain the intracranial hemorrhage (ICH) transformation. Symptomatic intracranial hemorrhage (sICH) was diagnosed according to the European Cooperative Acute Stroke Study III criteria [12]. All procedures and medical care were carried out in accordance with current guidelines and regulations [1]. Informed consents were obtained from the participants entering the stroke registry, and this analysis was approved by the Ethics Committee of Nanjing First Hospital.

### Statistical analysis

The baseline characteristics, procedure details, and clinical outcomes were compared between the three annual groups. Continuous data were presented as mean ± standard deviation or median (interquartile range) and categorical data as number (percentage). Metric and ordinal variables were analyzed by one-way ANOVA and Kruskal–Wallis test, respectively, while frequencies were compared using the chi-square test based on the linear-by-linear association. Next, we performed a logistic regression analysis to study the potential independent differences of successful recanalization, good outcome, sICH, and mortality over the 3 years. The first-year group was analyzed as a reference. Age, sex, and variables with *P* < 0.1 in univariate analysis were adjusted in multivariate models. The NIHSS score and puncture-to-recanalization time indicated successful recanalization; atrial fibrillation, creatinine, glucose, NIHSS score, stroke subtypes, puncture-to-recanalization time, additional interventions, and recanalization status were for good outcome; platelet counts, NIHSS score, onset-to-recanalization time, and recanalization status for sICH; creatinine, glucose, baseline systolic blood pressure, NIHSS score, infarct circulation, stroke subtypes, onset-to-recanalization time, number of device passes, and recanalization status indicated mortality. Statistical analyses were performed using Statistical Package for the Social Sciences (SPSS) version 20.0 (SPSS Inc., Chicago, IL, USA), and *P* < 0.05 indicated statistical significance.

## Results

A total of 222 patients were included in the present study; of these, 50 patients received endovascular treatment in the first year, 68 in the second year, and 104 in the third year. The average age of the cohort was 70.9 ± 11.9 years, and 64.9% was male. The median NIHSS score was 14. Of these patients, 81.1% had anterior, while 18.9% had posterior circulation infarction. The baseline characteristics of the patients are listed in Table 1. The discrepancies were detected among groups with respect to the history of stroke (8% vs. 19.1% vs. 24.0%, *P* = 0.021), serum creatinine [77 (64–102) vs. 77 (63–96) vs. 69 (58–85) μmol/L, *P* = 0.036], onset-to-door time [91 (38–180) vs. 145 (65–206) vs. 149 (77–283) min, *P* = 0.009], recruited beyond 6 h (2% vs. 2.9% vs. 7.3%, *P* = 0.001) and prior intravenous thrombolysis (56.0% vs. 64.7% vs. 40.4%, P = 0.021), while other variables were similar between different years.

**Table 1:** Baseline characteristics of patients in the three years

	Total (*n* = 222)	1st year (*n* = 50)	2nd year (*n* = 68)	3rd year (*n* = 104)	*P*
Age (years)	70.9 ± 11.9	69.5 ± 11.4	70.8 ± 13.4	71.7 ± 11.2	0.557
Sex, male	144 (64.9%)	31 (62.0%)	45 (66.2%)	68 (65.4%)	0.728
Medical history					
Hypertension, n (%)	163 (73.4%)	36 (72.0%)	46 (67.6%))	81 (77.9%))	0.308
Diabetes, n (%)	42 (18.9%)	5 (10.0%)	17 (25.0%)	20 (19.2%)	0.305
Atrial fibrillation, n (%)	113 (50.9%)	26 (52.0%)	30 (44.1%)	57 (54.8%)	0.555
Prior stroke, n (%)	42 (18.9%)	4 (8.0%)	13 (19.1%)	25 (24.0%)	0.021
Laboratory examination					
Platelet count, × 10^9^/L	175 (142–225)	176 (142–251)	175 (142–220)	177 (144–215)	0.604
INR, %	1.0 ± 0.1	1.0 ± 0.1	1.1 ± 0.2	1.0 ± 0.1	0.087
Serum creatinine, μmol/L	74 (61–93)	77 (64–102)	77 (63–96)	69 (58–85)	0.036
Glucose, mmol/L	6.5 (5.4–7.7)	6.3 (5.1–7.7)	6.8 (5.2–7.6)	6.5 (5.5–7.6)	0.511
Baseline SBP, mmHg	142 ± 21	145 ± 20	138 ± 23	144 ± 20	0.099
Baseline DBP, mmHg	87 ± 15	87 ± 12	84 ± 15	88 ± 15	0.212
Baseline NIHSS score	14 (11–19)	15 (12–18)	14 (9–18)	15 (11–20)	0.406
Infarct circulation					0.417
Anterior, n (%)	180 (81.1%)	42 (80.0%)	56 (82.4%)	82 (78.8%)	
Posterior, n (%)	42 (18.9%)	8 (16%)	12 (17.6%)	22 (21.2%)	
Stroke subtypes					0.955
LAA	96 (43.2%)	22 (44.0%)	29 (42.6%)	45 (43.3%)	
CE	108 (48.6%)	24 (48.0%)	33 (48.5%)	51 (49.0%)	
SOE	2 (0.9%)	1 (2.0%)	1 (1.5%)	0 (0%)	
SUE	16 (7.2%)	3 (6.0%)	5 (7.4%)	8 (7.7%)	
Onset to door time, min	132 (65–217)	91 (38–180)	145 (65–206)	149 (77–283)	0.009
recruited beyond 6 h, n (%)	21 (9.4%)	1 (2%)	2 (2.9%)	18 (7.3%)	0.001
Intravenous thrombolysis, n (%)	114 (51.4%)	28 (56.0%)	44 (64.7%)	42 (40.4%)	0.021

*Abbreviation*: *INR* international normalized ratio, *SBP* systolic blood pressure, *DBP* diastolic blood pressure, *NIHSS* National Institutes of Health stroke scale, *LAA* large artery atherosclerosis, *CE* cardiac embolism, *SOE* stroke of other determined etiology, *SUE* stroke of undetermined etiology

**Fig. 1:**
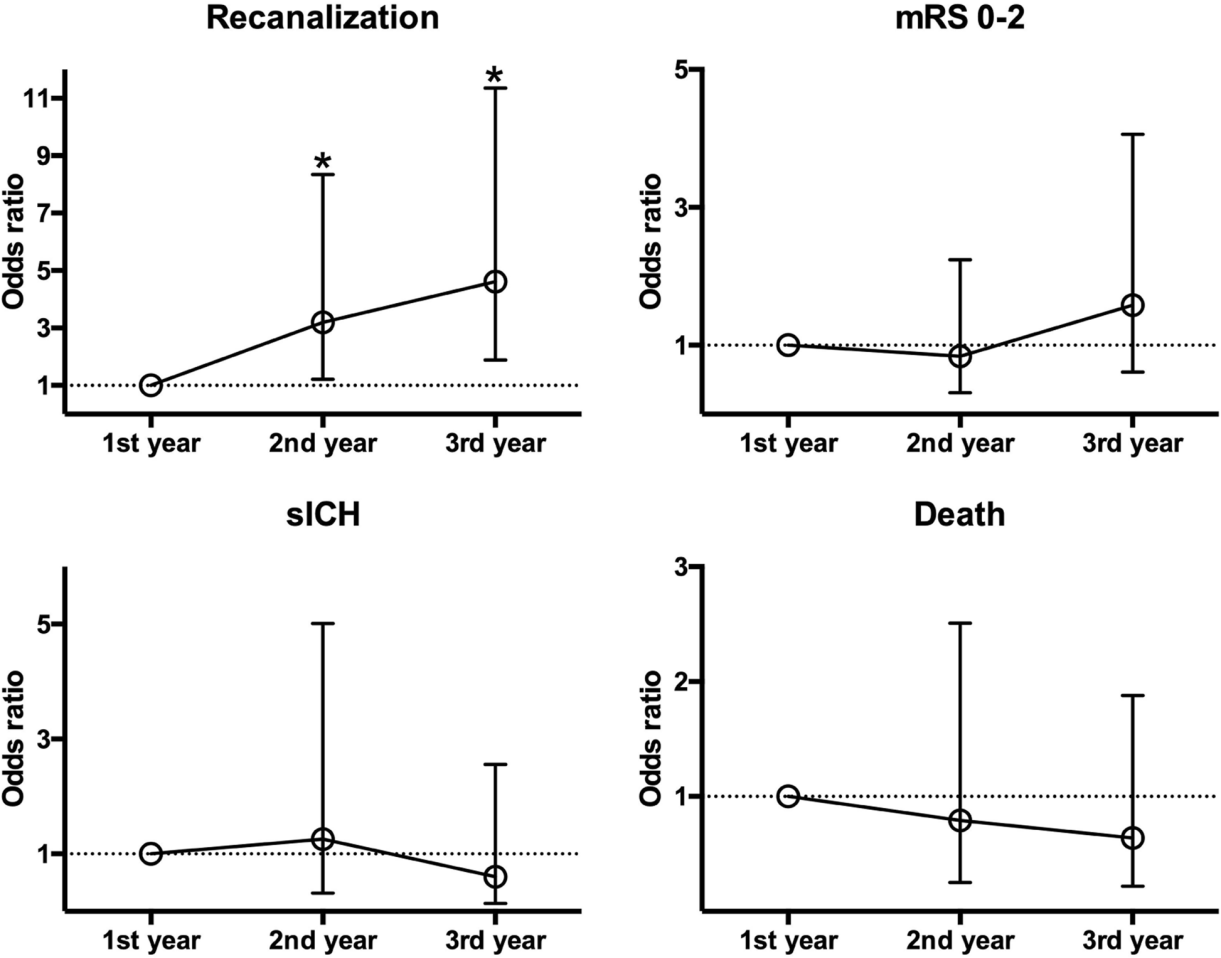
Adjusted odds ratios (midpoints) and 95% confidence intervals (error bars) derived from Logistic analysis for successful recanalization and clinical outcomes after mechanical thrombectomy categorized by years. Single asterisk (*) above the error bars represents the statistical significance (*P* < 0.05) between the annual group with the first-year group as reference. mRS indicates modified Rankin scale; and sICH, symptomatic intracranial hemorrhage

Details of endovascular treatment procedures are shown in Table 2. A significant decrease was observed in the door-to-puncture time [152 (123–189) vs. 119 (85–149), *P* < 0.001] and door-to-recanalization time [240 (185–280) vs. 194 (165–227), P < 0.001] from the first year to the second year. Compared to the patients of the first year, those of the second acquired successful recanalization (62.0% vs. 86.8%, *P* = 0.002). Furthermore, an increasing trend of good outcomes (mRS 0–2: 34.0% vs. 36.8% vs. 41.3%, *P* = 0.357) and a descending trend in intracranial hemorrhage (50.0% vs. 44.8% vs. 35.0%, *P* = 0.062), sICH (12.0% vs. 9.0% vs. 5.8%, *P* = 0.183), and mortality (24.0% vs. 17.6% vs. 18.3%, *P* = 0.462) was observed from the first to the third year. Logistic regression analysis showed that successful recanalization rates independently increased in the second year (OR: 3.19, 95% CI: 1.21–8.34, *P* = 0.020) and third year (OR: 4.62, 95% CI: 1.88–11.35, *P* = 0.001) as compared to the first year (Table 3 and Fig. 1).

**Table 2:** Characteristics of thrombectomy procedures and outcomes in the three years

	Total (*n* = 222)	1st year (*n* = 50)	2nd year (*n* = 68)	3rd year (*n* = 104)	*P*	P (1st vs.2nd)	P (2nd vs.3rd)
Door to groin puncture time (min)	122 (92–155)	152 (123–189)	119 (85–149)	106 (84–135)	< 0.001	< 0.001	0.507
Puncture to first recanalization time (min)	66 (51–102)	70 (53–119)	68 (54–103)	60 (45–79)	0.033	0.063	0.079
Door to first recanalization time (min)	197 (163–240)	240 (185–280)	194 (165–227)	177 (145–218)	< 0.001	< 0.001	0.111
Onset to first recanalization time (min)	340 (279–428)	357 (294–420)	352 (285–494)	311 (256–378)	0.005	0.073	0.005
Number of device passes	2 (1–3)	2 (1–4)	1 (1–2)	2 (1–3)	0.013	0.016	0.076
Additional interventions^a^	37 (16.7%)	9 (18.0%)	14 (20.6%)	14 (13.5%)	0.367	0.727	0.217
First pass effect	36 (16.2%)	3 (6.0%)	12 (17.6%)	21 (20.2%)	0.035	0.062	0.679
mTICI score					< 0.001	0.002	0.736
3	107 (48.2%)	15 (30.0%)	36 (52.9%)	56 (53.8%)			
2b	74 (33.3%)	16 (32.0%)	23 (33.8%)	35 (33.7%)			
2a	24 (10.8%)	11 (22.0%)	5 (7.4%)	8 (7.7%)			
1	9 (4.1%)	3 (6.0%)	2 (2.9%)	4 (3.8%)			
0	8 (3.6%)	5 (10.0%)	2 (2.9%)	1 (1.0%)			
Successful recanalization	181 (81.5%)	31 (62.0%)	59 (86.8%)	91 (87.5%)	0.001	0.002	0.888
mRS 0–2 at 90 days	85 (38.3%)	17 (34.0%)	25 (36.8%)	43 (41.3%)	0.357	0.758	0.549
Any ICH^b^	91 (41.4%)	25 (50.0%)	30 (44.8%)	36 (35.0%)	0.062	0.577	0.200
sICH ^b^	18 (8.2%)	6 (12.0%)	6 (9.0%)	6 (5.8%)	0.183	0.593	0.438
Death at 90 days	43 (19.4%)	12 (24.0%)	12 (17.6%)	19 (18.3%)	0.462	0.399	0.918

*Abbreviations*: *mTICI* modified thrombolysis in cerebral infarction, *mRS* modified Rankin scale, *ICH* intracranial hemorrhage, *sICH* symptomatic intracranial hemorrhage

^a^Additional interventions included aspiration, intra-arterial thrombolysis, balloon dilation, and stent implantation

^b^Any ICH and sICH were assessed in 220 patients while 1 patient in the 2nd year and 1 patient in the 3rd year died before repeated imaging

**Table 3:** Logistic analysis of successful recanalization and clinical outcomes

	Crude OR (95% CI)	*P*	Adjusted OR (95% CI)	*P*
Successful recanalization				
1st year	(reference)		(reference)	
2nd year	4.02 (1.63–9.93)	0.003	3.19 (1.21–8.34)	0.020
3rd year	4.29 (1.90–9.69)	< 0.001	4.62 (1.88–11.35)	0.001
mRS 0–2 at 3 months				
1st year	(reference)		(reference)	
2nd year	1.13 (0.53–2.43)	0.757	0.84 (0.31–2.24)	0.728
3rd year	1.37 (0.68–2.77)	0.382	1.58 (0.61–4.06)	0.345
sICH				
1st year	(reference)		(reference)	
2nd year	0.72 (0.22–2.39)	0.592	1.26 (0.32–5.01)	0.747
3rd year	0.45 (0.14–1.49)	0.192	0.60 (0.14–2.56)	0.493
Death				
1st year	(reference)		(reference)	
2nd year	0.68 (0.28–1.67)	0.398	0.79 (0.25–2.51)	0.687
3rd year	0.71 (0.31–1.60)	0.408	0.64 (0.22–1.88)	0.415

*Abbreviations*: *mRS* modified Rankin scale, *sICH* symptomatic intracranial hemorrhage

## Discussion

In this study, significant improvements were detected in the procedural delays and successful recanalization rates with accumulating experience. We also found an increasing trend of good outcomes and a decreasing trend of any intracranial hemorrhage and sICH post-procedures, albeit without statistical significance. After balanced for stroke severity and procedure duration, 3.19- and 4.62-fold of recanalization rates were acquired in the second and third year as compared to the first year.

Several randomized controlled trials have verified the benefits of thrombectomy. Although in our research, the onset to recanalization time was distinctly longer than that in ESCAPE trial [241 (176–359)], which may be related to the latter recruited more patients (72.7%) in the time window for intravenous thrombolysis, the recanalization rate (72.4%) was inferior to ours [13]. In DEFUSE 3 trial, puncture to reperfusion time [38(26–59)] was obviously less than ours and recanalization rate (76%) was slightly lower [14]. Even though revascularization rate in our center was superior to some trials, procedure time still could be shortened which need operators to be more proficient in the skill.

A minimum volume was set for certification of thrombectomy-capable stroke centers as practice makes the procedure perfect [15]. However, the major issue remained what was a reasonable number [16]. An early retrospective survey studied 442 patients treated with endovascular therapy. A shorter admission delay, higher reperfusion rates, and better clinical outcomes were observed at high-volume centers (≥50 procedures annually) than low-volume centers (< 50 procedures annually) [17]. In a national database study, the threshold of high-volume centers was ≥35.2 procedures per year, and the results demonstrated significantly reduced mortality in patients transferred to high-volume centers than directly admitted to the low-volume centers [3]. In the current study, angiographic outcomes improved annually continuously; also, the volume was beyond the criterion of high-volume in the above studies. The current results indicated that cumulative center experience might lead to better outcomes and supported the centralization of mechanical thrombectomy for large vessel occlusion stroke [3, 18].

Only a few studies referred to the cumulative experience on thrombectomy, and hence, the discrepancy was observed. Eesa et al. found that with experience accumulating, CT to recanalization time dramatically reduced owe to the improvement in the time from first stent deployment to recanalization. It implied that the learning curve exists in the efficient use of the Solitaire stent [19]. Weyland et al. affirmed that interventionalist’s experience tremendously affected procedure time and marked difference occurred in all outcome variables between the first 25 cases and the 26th to 50th cases [20]. In our study, we also could see considerable decrease in puncture-to-recanalization time between the first year and the last 2 years, which could be attributed to the operators gradually mastered the device. Sheth et al. analyzed the data of patients in the initial roll-in period and subsequent randomized phases of the Solitaire With the Intention For Thrombectomy (SWIFT) trial; the operators had no prior experience of using these devices. The results of the reperfusion rate (55% vs. 61%), adverse event (13% vs. 9%), and good neurological outcome (63% vs. 58%) were similar between the two periods, thereby suggesting a rapid learning curve for Solitaire stent retriever therapy [8]. The absence of a continuous improvement in thrombectomy might be possibly due to the limited number of procedures. There were only 31 and 58 patients in the first and second periods, respectively, and 50 procedures had been completed in the first year in this study, implying that a minimum volume might be warranted for a cumulative improvement effect. Furthermore, all participating centers in the SWIFT study were required to be familiar with early retriever devices, and our center did not have any prior experience of mechanical thrombectomy, which might also contribute to the difference in the results. The current findings were similar to those of another multicenter study by Kim et al. [9] consisting of 955 patients classified into 5 groups based on the consecutive number at each hospital. The results showed that the cumulative case volume group was positively correlated to the recanalization rate and good outcome and negatively correlated with sICH and mortality. However, patients were enrolled from different hospitals, i.e., from almost every center in the first group, while patients in the last group were only collected from high-volume hospitals. This significant correlation might be partially attributed to the different performances of high-volume and low-volume hospitals but not cumulative experience. In the current study, all procedures were performed by the fixed operators in a single-center, and the differences might be due to the diverse experience accumulation.

Nevertheless, the present study had some limitations. First, as a retrospective analysis of a single-center, selection bias is inevitable, and hence, our findings should be modestly referred by the other centers. Second, Just as Weyland et al. illustrated that interventionalist’s performance improved with operation volume increasing. Different operators’ experience could influence surgery duration and successful recanalization rates. But we could not separate the three interventionalists because our stroke register database lacked specific operator records and most procedures were performed by the three interventionalists together. Third, patients in the last 2 years with a prior stroke had a prolonged duration from onset to admission than the first year in the current study, implying putative differences in the selection of patients and treatment decision-making during the phase. In recent years, advanced imaging including perfusion has been widely used before endovascular treatment [21], especially after the publishment of the DAWN and DEFUSE3 trial results, thus we can see significant difference in the rate of patients recruited beyond 6 h in three groups. These improvements or differences in selection for treatment might also confound the association of experience and effect, which should be taken into consideration while comparing thrombectomy outcomes during different years or between high-volume and low-volume centers. Fourth, the median baseline NIHSS score (14 for the whole 3 years and 15 on the 3rd year) was modestly lower than that in the previous clinical trials, which was 17 in the meta-analysis [22]. Some extremely severe patients might be excluded from thrombectomy in our center, which might influence our results. Nevertheless, the severity of stroke in our study was comparable to that of a national registry [23]; thus, could reflect the real characteristics of patients in clinical practice.

## Conclusions

We found that cumulative experience could increase the rate of successful recanalization by years, which in turn, would improve the prognosis in the future studies carried out in multiple centers using a large sample size. The current findings endorsed a clinical benefit of centralization strategies of mechanical thrombectomy.

## Data Availability

The datasets used and/or analysed during the current study are available from the corresponding author on reasonable request.

## Abbreviations

NIHSS: National Institute of Health Stroke Scale
mTICI: Modified thrombolysis in cerebral infarction
mRS: Modified Rankin scale
sICH: Symptomatic intracranial hemorrhage
SPSS: Statistical Package for the Social Sciences
SWIFT: Solitaire With the Intention For Thrombectomy
